# Compressibility of porous TiO_2_ nanoparticle coating on paperboard

**DOI:** 10.1186/1556-276X-8-444

**Published:** 2013-10-25

**Authors:** Milena Stepien, Jarkko J Saarinen, Hannu Teisala, Mikko Tuominen, Janne Haapanen, Jyrki M Mäkelä, Jurkka Kuusipalo, Martti Toivakka

**Affiliations:** 1 Laboratory of Paper Coating and Converting, Center for Functional Materials, Åbo Akademi University, Porthansgatan 3, Turku FI-20500, Finland; 2 Paper Converting and Packaging Technology, Department of Materials Science, Tampere University of Technology, P.O. Box 589, Tampere FI-33101, Finland; 3 Aerosol Physics Laboratory, Department of Physics, Tampere University of Technology, P.O. Box 692, Tampere FI-33101, Finland

**Keywords:** Nanoparticles, Compressibility, Wettability, Liquid flame spray process

## Abstract

Compressibility of liquid flame spray-deposited porous TiO_2_ nanoparticle coating was studied on paperboard samples using a traditional calendering technique in which the paperboard is compressed between a metal and polymer roll. Surface superhydrophobicity is lost due to a smoothening effect when the number of successive calendering cycles is increased. Field emission scanning electron microscope surface and cross‒sectional images support the atomic force microscope roughness analysis that shows a significant compressibility of the deposited TiO_2_ nanoparticle coating with decrease in the surface roughness and nanoscale porosity under external pressure.

**PACS:**

61.46.-w; 68.08.Bc; 81.07.-b

## Background

Nanoparticles exhibit extraordinary electronic, optical, and mechanical properties compared to bulk materials. This is due to two facts: first, nanoparticles have a large surface-to-volume ratio, i.e., a large number of atoms are located on the surface with distinct contribution to the free energy; second, quantum confinement manifests in small scale. For example, the color of nanoparticles can be varied over the whole visible spectrum simply by controlling the size and morphology of silver nanosphere lithography [1] or the size of semiconductor quantum dots such as CdS [2]. Nanosized TiO_2_ particles have been applied in various industries ranging from sunscreen cosmetics [3] and whitening paint pigments [4] to catalyst supports [5], dye-sensitized solar cells [6], and self-cleaning surfaces via photocatalytic activity [7]. TiO_2_ can be found in four different crystalline forms: anatase, rutile, brookite, and akaogiite - a dense, high-pressure phase of TiO_2_[8-10]. The crystalline structure of TiO_2_ particles plays a crucial role, for example, in dye-sensitized solar cells, which require anatase phase [11,12].

We have recently demonstrated controlled wettability from superhydrophobic to highly hydrophilic surfaces on TiO_2_ nanoparticle-coated paperboard by liquid flame spray (LFS) deposition [13]. It is noteworthy that superhydrophobicity is only observed on paper and paperboard whereas TiO_2_ nanoparticle deposition by LFS on aluminum foil resulted in a slightly hydrophilic surface [14]. Superhydrophobicity on a paperboard originates from the nanoscale roughness with the organic components, typically binders used in papermaking that will evaporate during the LFS deposition with surface temperatures of 100°C to 300°C on paperboard surface 0.5 m after the flame [15]. These volatile organic compounds condense into a thin carbonaceous layer on deposited TiO_2_ nanoparticles.

Flame-based methods for nanoparticle deposition have been investigated since the 1980s [16-21]. In the LFS process, a liquid precursor is fed into a high-temperature flame in which the precursor is atomized into small droplets that evaporate in the flame. The precursor material gas decomposes and nucleates forming nanoparticles that can be collected on a moving web. LFS is suitable for deposition of various metal and metal oxide nanoparticles with a relatively narrow and controllable size distribution of nanoparticles with diameters from 2 to 200 nm [20]. The morphology of the deposited nanoparticles can be controlled via process parameters including gas and precursor feed rates, precursor concentration, distance of the substrate from the burner, and deposition time (web speed) [22].

In this article, we investigate the compressibility of such LFS-deposited TiO_2_ nanoparticle coating on paperboard by calendering. Calendering is a traditional surface finishing technique widely used in the paper industry to give the paper surface a smoother and glossier look [23]. In calendering nip, paperboard web is compressed between rolls with controllable temperature, pressure, nip time (web speed), and nip roll materials. Compressibility of the nanoparticle coating will affect surface properties such as wettability. Individual nanoparticle compressibility has been studied [24-26] under high-pressure by X-ray diffraction. However, as far as the authors know, a systematic study of porous nanoparticle coating compressibility has not been presented until now.

## Methods

**Figure 1:**
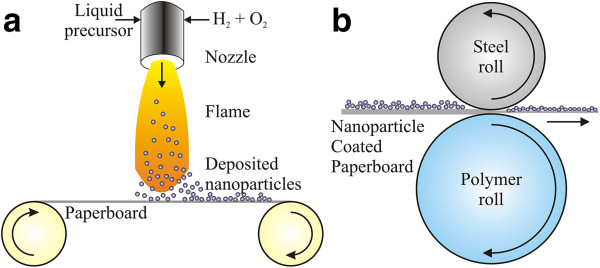
**TiO**_**2 **_**nanoparticle deposition and compression of nanoparticle-coated paperboard. (a)** TiO_2_ nanoparticle deposition on paperboard surface by the LFS process. **(b)** Compression of nanoparticle-coated paperboard by calendering with hard metal and soft polymer roll calender.

The reference substrate is a commercial double pigment-coated paperboard (200 g/m^2^, Stora Enso, Sweden) manufactured with an online coating process that was used as a substrate for the TiO_2_ LFS nanoparticle deposition. A schematic picture of the LFS deposition process is shown in Figure 1a. Nanoparticle-coated samples were prepared in a roll-to-roll process using coating and laminating pilot line at the Tampere University of Technology (Tampere, Finland) with a constant web speed of 50 m/min. Titanium(IV) isopropoxide (TTIP; 97% pure, Aldrich, St. Louis, MO, USA) dissolved in isopropanol (IPA) was used as a precursor for the TiO_2_ nanoparticle coatings with a metal ion concentration of 50.0 mg/ml. The precursor was fed into a spray nozzle with a rate of 12.0 ml/min fixed at 6-cm distance from the moving paperboard substrate. Hydrogen (50 l/min) and oxygen (15 l/min) were used for combustion gases in the process.

The compressibility of TiO_2_ nanoparticle-coated paperboard surfaces was investigated by calendering in which the paperboard is compressed between two rolls as shown in Figure 1b. Calendering is a well-known surface finishing technique widely used in papermaking. In our case, we use a soft roll/hard roll calender (DT Laboratory Calender, DT Paper Science Oy, Turku, Finland) with a lineload of 104 kN/m and a temperature of 60°C. The samples were treated with the same parameters in successive calendering nips with the nanoparticle-coated surface always facing the steel roll to prevent nanoparticle adhesion to the polymer roll. A schematic illustration of the calender is presented in Figure 1b.

Surface chemistry was studied with water contact angle measurements performed using the commercial contact angle goniometer KSV CAM 200 (KSV Instruments Ltd., Helsinki, Finland) with an automatic dispenser and motorized stage. The images of the droplets were captured by a digital CCD camera with a 55-mm-zoom microscope lens with a blue LED light source and analyzed with the KSV CAM software. The standard deviation of the contact angle (CA) measurements was approximately ±3°. Contact angles of the Milli-Q (Millipore, Billerica, MA, USA, resistivity 18.2 MΩ) purified water was measured in air in ambient conditions (room temperature 23°C ± 1°C and relative humidity 30% ± 5%) after 2 s of the droplet application. The volume of the droplets was approximately 2.0 μL, and the reported CA values are mean values of three individual measurements.

The TiO_2_ nanoparticle-coated paperboard surface was exposed to UVA light (Bluepoint 4 ecocure, Hönle UV Technology, Gräfelfing, Germany) with a central wavelength of 365 nm using a filter for 320 to 390 nm. A constant intensity of 50 mW/cm^2^ was applied for 30 min that converted the initially superhydrophobic surface to a highly hydrophilic one.

The scanning electron microscopy (SEM) imaging of the samples was performed using a field emission scanning electron microscope (FE-SEM; SU 6600, Hitachi, Chiyoda-ku, Tokyo, Japan) with an in-lens detector. All samples were carbon-coated to obtain conductivity. The secondary electron (SE) imaging mode was used for topographical imaging with a magnification of ×50,000 and ×5,000 with an accelerating voltage of 2.70 kV and a working distance of 4 to 5 mm. Cross sections of the TiO_2_ nanoparticle-coated samples were prepared using an Ilion+ Advantage-Precision Cross-Section System (Model 693, Gatan Inc., Pleasanton, CA, USA). One cross section was milled for each calendered sample with an argon broad ion beam using an accelerating voltage of 5 kV for 150 min. The paper samples were platinum-coated before the cutting to improve heat exchange and to reduce heat damage at the cutting area.

An atomic force microscope (AFM; NT-MDT NTEGRA Prima, Moscow, Russia) was used for further surface characterization. Gold-coated, reflective probes (NSG10) were used with an intermediate spring constant *k* = 11.5 N/m, a maximum tip radius of curvature of 10 nm, and a resonance frequency of 190 to 325 kHz (Europe MicroMasch, Tallinn, Estonia). Images were captured using the tapping mode at ambient conditions (room temperature 24°C ± 1°C and relative humidity 38% ± 5%). After landing with tip on the sample surface, a damping ratio (*A*_sp_/*A*_0_) of 0.5 to 0.6 and a line frequency of 0.25 to 0.6 Hz were optimized for imaging. The AFM was placed on a vibration isolation table (TS-150, Table Stable, Zwillikon, Switzerland) to eliminate external vibrational noise. Image processing and root-mean-square (RMS) roughness *S*_q_ calculations were carried out using the scanning probe image processor program (SPIP™, Image Metrology A/S, Hørsholm, Denmark). Before calculation, images were plane-corrected and the ISO 11562 Gaussian profile filter was implemented.

## Results and discussion

**Figure 2:**
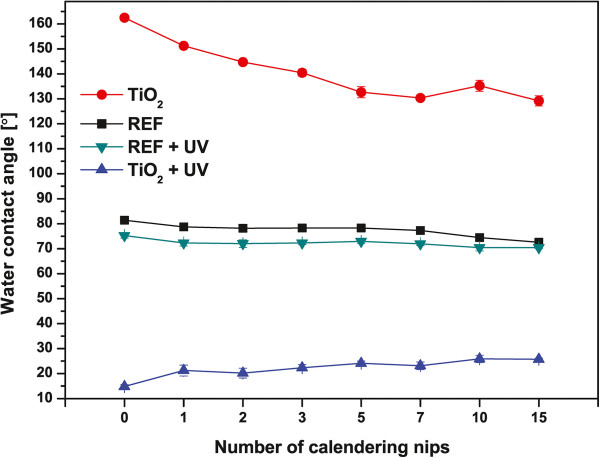
**Water contact angles as a function of the number of calendering nips.** For TiO_2_ nanoparticle-coated and the reference paperboard. The values are taken after 2 s of the droplet application on the surface.

TiO_2_ nanoparticle coatings on paperboard exhibit superhydrophobicity (water contact angle above 160°) that can be converted into a highly hydrophilic surface (water contact angle below 20°) by ultraviolet (UV) illumination via the photocatalytic activity of TiO_2_ as presented in Figure 2. The crystalline form of the LFS-deposited TiO_2_ nanoparticles is mainly anatase [22], analyzed from the TEM diffraction pattern. UV light induces free radicals and photocatalytic oxidation that change the surface chemistry of nanoparticles from hydrophobic to hydrophilic. In our previous study [13], we used X-ray photoelectron spectroscopy (XPS) to study the mechanisms of such wettability conversion: after the UV irradiation, increased values of both O/C and O/Ti ratios were observed. This corresponds to the increased amount of hydroxyl groups on the outermost TiO_2_ nanoparticle surface. Furthermore, our time-of-flight secondary ion mass spectrometry (ToF-SIMS) analysis [14] was in agreement with the XPS results with decreased relative amounts of hydrocarbons after the UV irradiation. The surface superhydrophobicity can be recovered by a heat treatment. After the heat treatment, the O/C and O/Ti ratios decreased, and the highly resolved spectra of O 1*s* verified the decreased amount of oxygen related to the hydroxyl groups [13]. A similar change is observed in the ToF-SIMS spectra [14] with increased relative amounts of hydrocarbon chains originating from the volatile organic compounds used in the base paper substrate. We have previously shown that surface wettability can be alternated between wetting and non-wetting states for several cycles, and the observed changes in wettability correlate well with the changes in the surface chemistry of the TiO_2_ nanoparticle-coated surface [13,14].

It is well known that superhydrophobicity can only be observed on rough surfaces, i.e., both chemical and physical effects contribute to superhydrophobicity. Classical theories by Wenzel [27] and Cassie and Baxter [28] have been used to explain observed contact angles on rough substrates: on rough, hydrophobic surfaces, the water droplet resides mostly on air and thus exhibits very high contact angles. Shibuichi et al. [29,30] presented an elegant analysis of how apparent contact angles are affected by the surface roughness compared to a smooth surface. Here, in our study, the bulk compressibility of the reference paperboard has a minor effect on water contact angles whereas superhydrophobic TiO_2_ nanoparticle-coated paperboard supports the analysis by Shibuichi et al. [29,30]: increasing the number of calendering nips results in a decrease of the water contact angles on the hydrophobic side and increase on the hydrophilic side after the ultraviolet treatment in Figure 2. This is expected as adding the number of successive calendering nips will reduce surface roughness. The water contact angle is approximately 130° and 25° after 15 calendering nips for TiO_2_ nanoparticle-coated samples without and with UV treatment, respectively. This indicates that the TiO_2_ nanoparticles do not adhere to the steel calender roll but rather remain on the paperboard surface. Removal of the nanoparticles from the surface would bring the contact angles closer to those values of the reference paperboard in which the water contact angles are almost independent of both the number of calendering nips and the UV treatment.

**Figure 3:**
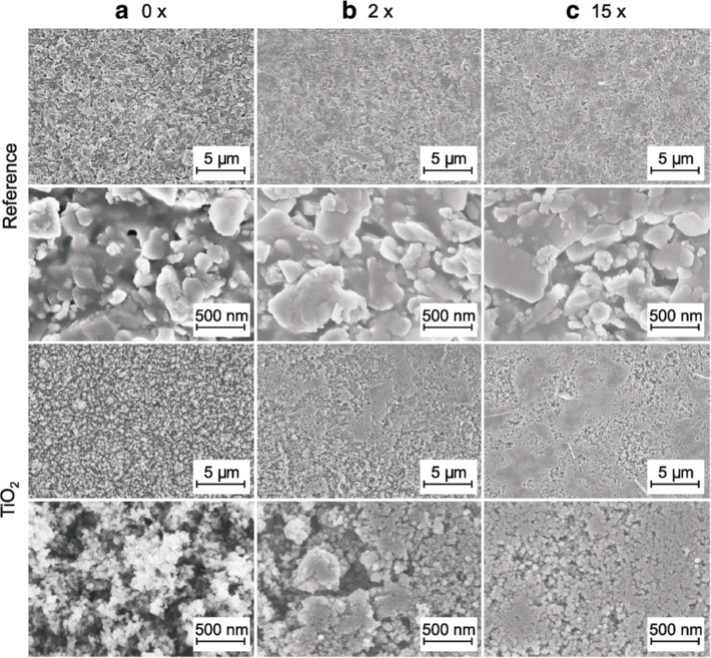
**FE-SEM images of the samples. (a)** Uncalendered sample and calendered samples **(b)** ×2 and **(c)** ×15 for reference paperboard and TiO_2_ nanoparticle-coated samples in low and high magnifications.

The surface of the reference paperboard was imaged using an FE-SEM showing mineral pigment particles (kaolin and calcium carbonate) immersed in an organic binder with pigment particle sizes in the range of microns as shown in Figure 3a. The high-magnification reference image displays the platy-like kaolin particles used in the pigment coating. The LFS coating of TiO_2_ nanoparticles results in a surface fully covered with nanoparticles as presented in the low-magnification image of Figure 3a, and the average nanoparticle diameter is approximately 20 to 40 nm as depicted from the high-resolution image of the LFS-coated TiO_2_ sample in Figure 3a. Calendering evens both reference and nanoparticle-coated paperboard surfaces. However, there is a more significant change in the morphology of the nanoparticle-coated sample as clearly seen in Figure 3b,c. High-magnification images of TiO_2_ nanoparticle coating in Figure 3b,c show that under compression nanoparticles start to cluster together forming large smooth areas. The size of these areas increases with the number of calendering nips. It is known from the literature that the compressibility of nanoparticles increases with decreasing particle size [24]. Even some structural transformations can take place in nanoscale that do not exist in macroscale [31]. The used pressures in the previous individual nanoparticle compressibility studies have been in the range of gigaPascal [24-26], which is several orders of magnitude higher than in our calendering nip. However, we are here studying the compressibility of the whole nanoporous TiO_2_ layer.

**Figure 4:**
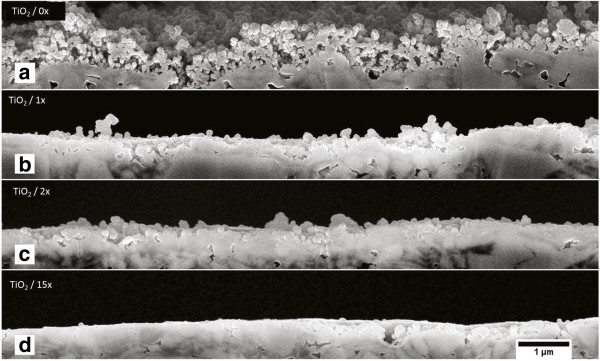
**FE-SEM cross-sectional images of the samples. (b)** Uncalendered sample and calendered samples **(b)** ×1, **(c)** ×2, and **(d)** ×15 calendering nips.

Changes in the thickness of the nanoparticle coating layer were estimated from FE-SEM cross-sectional images of the TiO_2_ nanoparticle-coated and calendered paperboard. The cross-sectional samples were prepared by broad ion beam milling technique using an argon ion beam, and the samples were carbon-coated before imaging. The uncalendered sample in Figure 4a shows a porous TiO_2_ nanoparticle coating with a thickness of approximately 600 to 700 nm. Even a single treatment in Figure 4b or double treatment in Figure 4c through the calendering nip significantly compresses the nanoparticle coating. Finally, the ×15 calendered sample in Figure 4d shows almost uniform surface characteristics along the imaged area. The porosity of the nanoparticle coating can also be estimated from the FE-SEM cross-sectional image: the nanoparticle coating thickness is approximately 600 nm with the deposition amount of 100 mg/m^2^ obtained from inductively coupled plasma mass spectrometry resulting in the average porosity of 95.7% for the TiO_2_ nanoparticle coating (using an anatase density of 3.89 g/cm^3^).

**Figure 5:**
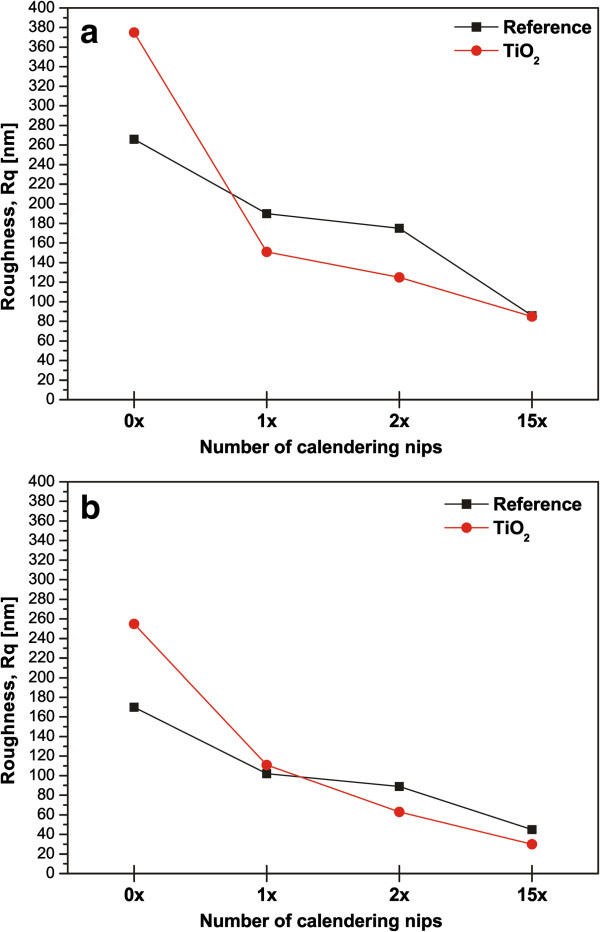
**AFM roughness analysis.** From image sizes of **(a)** 100 × 100 μm^2^ and **(b)** 20 × 20 μm^2^ as a function of the number of calendering nips.

Finally, we quantified the sample surface roughness using AFM. Images were captured in tapping mode in ambient conditions using a gold-coated tip having a surface radius of 10 nm. Two different image areas were analyzed: 100 × 100 and 20 × 20 μm^2^, shown in Figure 5a,b. Both image areas show that the TiO_2_ nanoparticle-coated sample has a higher RMS roughness *R*_q_ value than the reference paperboard before calendering. This is in agreement with our previous analysis [32]. Furthermore, even a single calendering reduces roughness values by more than 50% for nanoparticle-coated samples. The change in roughness values is significantly smaller for the reference paperboard. This is in agreement with the water contact angle results in Figure 1: the effect of roughness is less prevalent when the water contact angles are in the vicinity of 90°. Therefore, small changes in the surface roughness do not induce large changes in the water contact angle. We also examined the RMS roughness analysis as a function of the correlation length from the 20 × 20 μm^2^ AFM images. For the uncalendered TiO_2_ nanoparticle-coated sample, the RMS roughness decreases as the correlation length decreases. However, this behavior is different even for the single calendered sample as the RMS roughness is almost a plateau as a function of the correlation length excluding values smaller than 0.4 μm. This also confirms how the nanoporous coating layer compresses in the calendering nip.

## Conclusions

In summary, we have investigated the compressibility of TiO_2_ nanoparticle coatings on paperboard. Our analysis shows that the morphology of deposited nanoparticle coating undergoes a significant transition even in a single calendering cycle. The surface roughness values are reduced as expected, and nanoparticle coating shows a higher sensitivity for the compression than the reference paperboard. The compression will reduce superhydrophobicity as air pockets collapse in nanoporous TiO_2_ coating under compression as clearly observed from the SEM cross-sectional images. We believe that LFS-deposited nanoparticle coatings will find many applications in the future from controlled wettability to enhanced sensing in surface-enhanced Raman scattering. Understanding the stability of such nanoparticle coatings is crucial for reproducible and reliable performance of the functional coatings.

## Abbreviations

AFM: Atomic force microscopy; CA: Contact angle; CCD: Charge-coupled device; FE-SEM: Field emission scanning electron microscope; IPA: Isopropanol (or isopropyl alcohol); LED: Light-emitting diode; LFS: Liquid flame spray; RMS: Root-mean-square; SE: Secondary electron; SEM: Scanning electron microscopy; ToF-SIMS: Time-of-flight secondary ion mass spectrometry; TTIP: Titanium(IV) isopropoxide; UVA: Ultraviolet A; XPS: X-ray photoelectron spectroscopy.

## Competing interests

The authors declare that they have no competing interests.

## Authors’ contributions

MS, JJS, and MT (AAU) designed and planned the experiments. HT, MT (TUT), JH, JMM, and JK fabricated the nanoparticle-coated paperboard samples. MS conducted all the experiments and performed the data analysis. JJS wrote the manuscript. All authors read and approved the final manuscript.
